# Production and Physicochemical and Structural Characterization of a Hydrochar Derived from Agave Leaves and Tequila Vinasse with Potential Agricultural Applications

**DOI:** 10.3390/molecules31183173

**Published:** 2026-09-10

**Authors:** Dania Carolina Uvario-Macías, José Anzaldo-Hernández, Carlos Alberto Ramírez-Barragán, Fernando Santacruz-Ruvalcaba, Dulce Flores-Rentería, Juan Carlos Pizano-Andrade, Eduardo Salcedo-Pérez

**Affiliations:** 1 University Center for Biological and Agricultural Sciences (CUCBA), University of Guadalajara, Guadalajara-Nogales Highway, Km 15.5, Las Agujas, Zapopan 45020, Jalisco, Mexico; dania.uvario6755@alumnos.udg.mx (D.C.U.-M.); fernando.santacruz@academicos.udg.mx (F.S.-R.); pizanjc@gmail.com (J.C.P.-A.); 2 Department of Wood, Cellulose, and Paper (CUCEI), University of Guadalajara, Guadalajara-Nogales Highway, Km 15.5, Las Agujas, Zapopan 45020, Jalisco, Mexico; jose.anzaldo@academicos.udg.mx (J.A.-H.); carlos.ramirez@dmcyp.cucei.udg.mx (C.A.R.-B.); 3 SECIHTI-Cinvestav Saltillo Unit, Ramos Arizpe Industrial Park, Ramos Arizpe 25900, Coahuila, Mexico; yaahid.flores@cinvestav.edu.mx

**Keywords:** hydrothermal carbonization, tequila, waste, circular economy, plant biomass

## Abstract

The generation of agro-industrial waste necessitates the search for viable, sustainable alternatives for its utilization; therefore, Hydrothermal Carbonization (HTC) is proposed as a means to transform waste into carbonaceous products that can improve soil quality. To this end, conditions in the HTC process were evaluated for the production of hydrochar from tequila vinasse and *Agave tequilana* Weber leaves. Different reaction times (8, 12, and 16 h) and catalyst concentrations (0%, 5%, and 10%) of citric acid were evaluated at 200 °C, using fresh agave leaves and raw vinasse. The hydrochars were characterized using various instrumental analyses (XPS, FTIR, SEM-EDS, and XRF) to determine their structure, functional groups, and elemental composition. The highest yield was obtained at 8 h without a catalyst (44.6%), decreasing with increasing process time. The vinasse served effectively as an aqueous medium and natural catalyst, with its acidic pH (3.8), thereby eliminating the need for water and acid addition. With longer processing times, microspheres form within the hydrochar structure due to carbohydrate degradation. The hydrochar produced without a catalyst and with a shorter reaction time exhibited functional groups (carboxyl and hydroxyl) that enhance Cation Exchange Capacity (CEC), thereby increasing the contact surface area. Under the evaluated conditions, the HTC process is suitable for producing hydrochar from agave leaves and vinasse.

## 1. Introduction

Climate change has become a major global concern, driven by rising temperatures and greenhouse gas emissions. The agricultural, livestock, and fishing sectors, among others, must adopt new technologies to adapt to changing climate scenarios and to society’s growing demand for food [1].

In this context, agricultural producers must seek new farming methods to mitigate soil degradation and, primarily, prevent the release of gases throughout the production chain. Therefore, given the degradation of soil’s physical and chemical properties, the incorporation of organic amendments is a key strategy for restoring soil functionality [2]. Materials such as compost, green manure, crop residues, and manure act to improve the physicochemical and biological conditions of the soil environment [3]. In this context, carbonaceous materials such as biochar and hydrochar have recently gained significant relevance due to their specific physicochemical properties.

Hydrothermal carbonization (HTC) stands out as a technological alternative for the valorization of wet waste biomass. This thermochemical process occurs in an aqueous medium under anoxic conditions (absence of oxygen), at temperatures of 180 to 250 °C and autogenous pressures of 20 to 40 bar [4]. The reaction, which can last between 5 min and 16 h, generates a main solid product called hydrochar, and byproducts consisting of a liquid phase and a minor gaseous fraction [5,6]. To optimize this process, the use of catalysts (primarily acids) has been investigated to increase reaction yield and enhance the physicochemical properties of the resulting material [7]. It should be noted that both operating conditions and catalyst use are decisive factors in the final characteristics of the hydrochar. The rise in HTC is primarily driven by the need to process biomass with high moisture content, thereby reducing energy consumption and production costs compared to the pyrolysis process [4,8].

Thanks to its distinctive physicochemical properties, hydrochar has high potential as a soil conditioner. Physically, it promotes aggregate stability, modifies texture, and reduces bulk density, thereby increasing water-holding capacity [9]. Chemically, its surface functional groups can alter the pH and significantly increase Cation Exchange Capacity (CEC), improving nutrient retention [10]. Finally, from a biological perspective, hydrochar acts as a substrate and habitat for edaphic microbiota, promoting soil biological activity [11].

The versatility of the HTC process enables the recovery of a wide range of residual biomass, including critical agro-industrial byproducts in Mexico, such as agave leaves and tequila vinasse. Currently, the management of waste generated by the tequila industry poses a significant environmental challenge, due to the increase in volumes produced as the sector intensifies. It is estimated that the biomass of discarded leaves on a typical agave plot harvested after five years can reach approximately 90 t ha^−1^ of leaf waste, considering the cumulative production over the crop cycle. Likewise, vinasse generation is estimated based on the reported ratio of 8 L of vinasse per liter of tequila produced, using production data from the past five years [12,13]. Based on these estimates, an average of 4.235 billion liters of vinasse is generated per year, which constitutes a significant problem for the tequila industry due to limitations in its management and final disposal; it is important to evaluate whether the HTC process reduces parameters considered hazardous or polluting in the vinasse, making this process a viable option for treating these tequila industry wastes.

In this context, the objective of the present study was to evaluate the effects of different hydrothermal carbonization (HTC) process conditions on the yield and physicochemical properties of hydrochar produced from tequila vinasse and agave leaves, to determine its potential as a soil conditioner.

## 2. Results and Discussion

### 2.1. Chemical Characterization of the Source Materials

The chemical composition of the agave leaves used to produce hydrochar is described below. The lignin (12.53%) and holocellulose (79%) contents are consistent with those reported for *Agave tequilana* leaves (15.9% lignin and 69.9% holocellulose) [14,15]. The carbon content affects the energy density and yield of the hydrochar; therefore, the lignin and holocellulose content will be reflected in the amount of hydrochar obtained [16]. A high ash content negatively affects calorific value and yield and can also increase pollutant emissions during combustion [17]. Pasipanodya et al. [18] reported ash contents of 9.37% in walnut shells, while dos Santos et al. [19] reported that sugarcane bagasse contains 6.81% ash. Nitrogen benefits hydrochar when used as a soil conditioner, increasing its potential as a slow-release fertilizer [20].

Given the acidic pH of the vinasse (3.8) used as the feedstock, the catalyst (citric acid) can be omitted, as the vinasse acidifies the medium in the HTC process, enabling hydrolysis [21].

### 2.2. Hydrochar Yield

**Figure 1:**
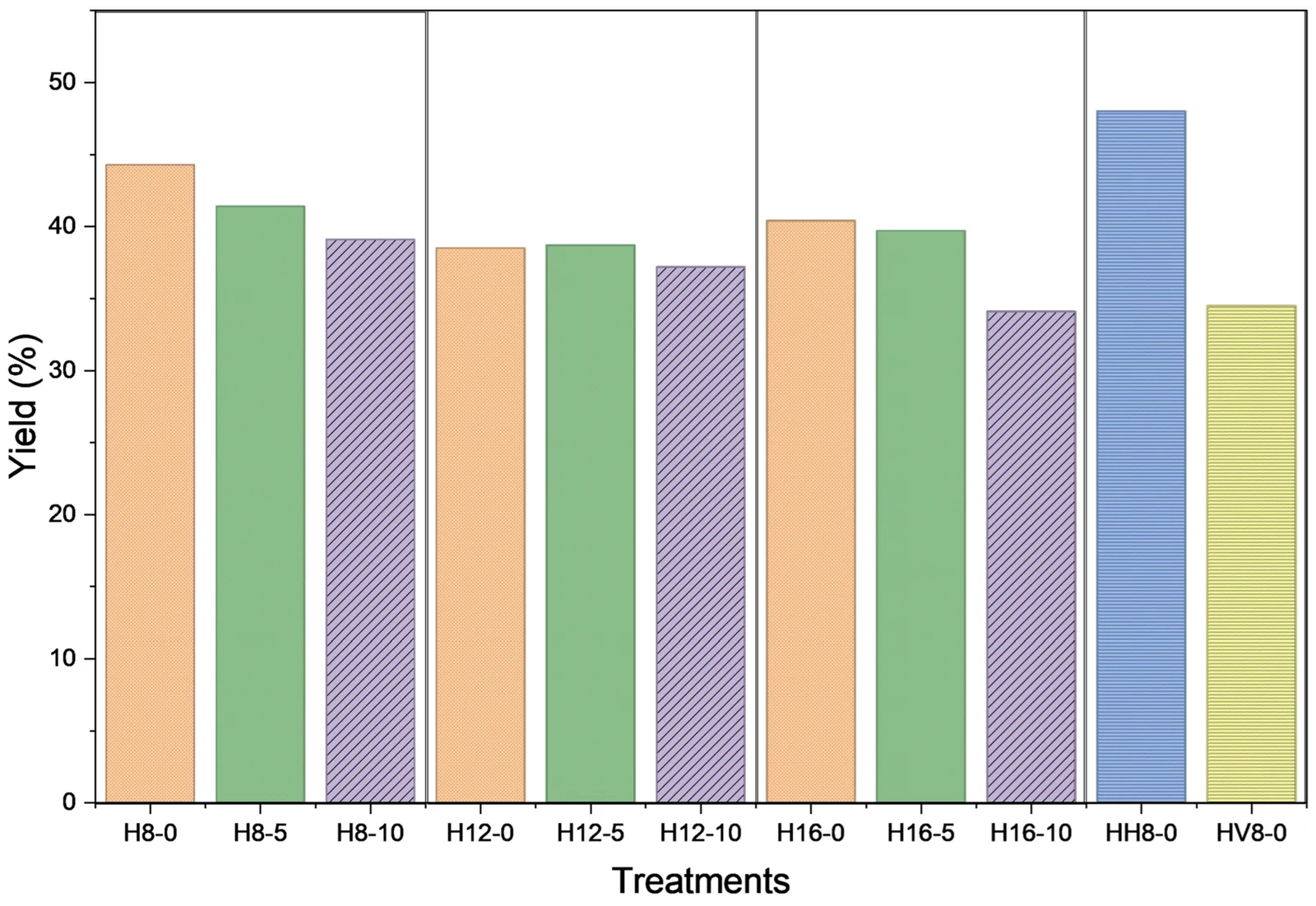
Hydrochar yield. Nomenclature: H (hydrochar), 8, 12, 16 (process hours), −0, −5, −10% (percentage of citric acid used in the HTC). HH8 (Leaf Hydrochar with 8 h). HV8 (Vinasse Hydrochar with 8 h).

Figure 1 shows differences in yield among the various treatments; the highest yield was observed in treatment HH8-0, in which only leaves and water were used for 8 h. On the other hand, the treatment using only vinasse (HV8-0) had the lowest yield. Regarding the yields obtained in the combined leaf and vinasse hydro-modules, where process time and catalyst concentration varied, differences were observed due to the different process conditions. A shorter processing time (8 h) and the absence of acid in the HH8-0 treatment resulted in the highest yields (Figure 1); this highlights its importance from a sustainability standpoint, as energy consumption is lower and water and catalyst use are replaced by vinasse.

Temperature, pH, catalyst use, and HTC retention time are key parameters for hydrochar yield [22]. In this study, significant differences were observed only between the H8-0 treatment (44.6%) and H16-10 (34.4%), confirming that longer processing times reduce yield, consistent with Khan et al. [23] who conclude that reaction intensity improves with a longer reaction time, as it has a similar e effect to temperature; if the time is short, the yield of hydrochar in the solid phase of the HTC will be higher.

On the other hand, when comparing HH8 with hydrochar produced from a mixture of leaves and vinasse, the yield decreased, attributed to the vinasse acting as a catalyst. Furthermore, no significant differences were observed when the citric acid concentration was varied during the HTC process. Thanks to the use of vinasse residues, water consumption in the process is unnecessary.

### 2.3. Chemical Characterization of the Hydrochars

Table 1 presents the chemical characteristics of the HTC hydrochars. Demineralized water was used as the reaction medium in the treatments with leaves only, yielding an initial pH of 5.3. For treatments utilizing leaves and vinasse, the initial pH was 3.8 (identical to the pH of vinasse alone). The final pH values of the treatments, presented in Table 1, were maintained within the range of 5.0 to 5.5, irrespective of the applied conditions. This is due to changes during the HTC process, primarily polymerization and condensation, as well as other mechanisms, such as molecular dehydration, that reduce the number of hydrogen ions, leading to the aromatization of the hydrochars [24].

The trace elements present in the different hydrochars originate from agave leaves, which absorb them from the soil due to the application of fertilizers, liming products, pesticides, etc. Although their availability is conditioned by certain chemical, mineral, and biological parameters of the soil, plants can absorb small amounts through their roots [25].

The presence of Co, As, Ag, Sb, Hg, Pb, Th, and U was not detected in any of the treatments. For K, Ca, Fe, and Mn, the levels are below those reported by Fornes et al. [26], whose hydrochar was based on forest biomass; Bento et al. [27], who used sugarcane bagasse and vinasse; and Torabian et al. [28], whose biomass was corn. The Ni content is close to that reported. Zn was not present in all hydrochars; however, in HH8-0, concentrations were higher than the normal limits for plants (±19.5) [29] but lower than those reported by Fornes et al. [26]. The Mo content of H12-0 and H16-0 exceeded the normal limits in plants, which could result in toxicity when applied to the soil [30].

### 2.4. Surface Functional Groups by FTIR and XPS Analysis

**Figure 2:**
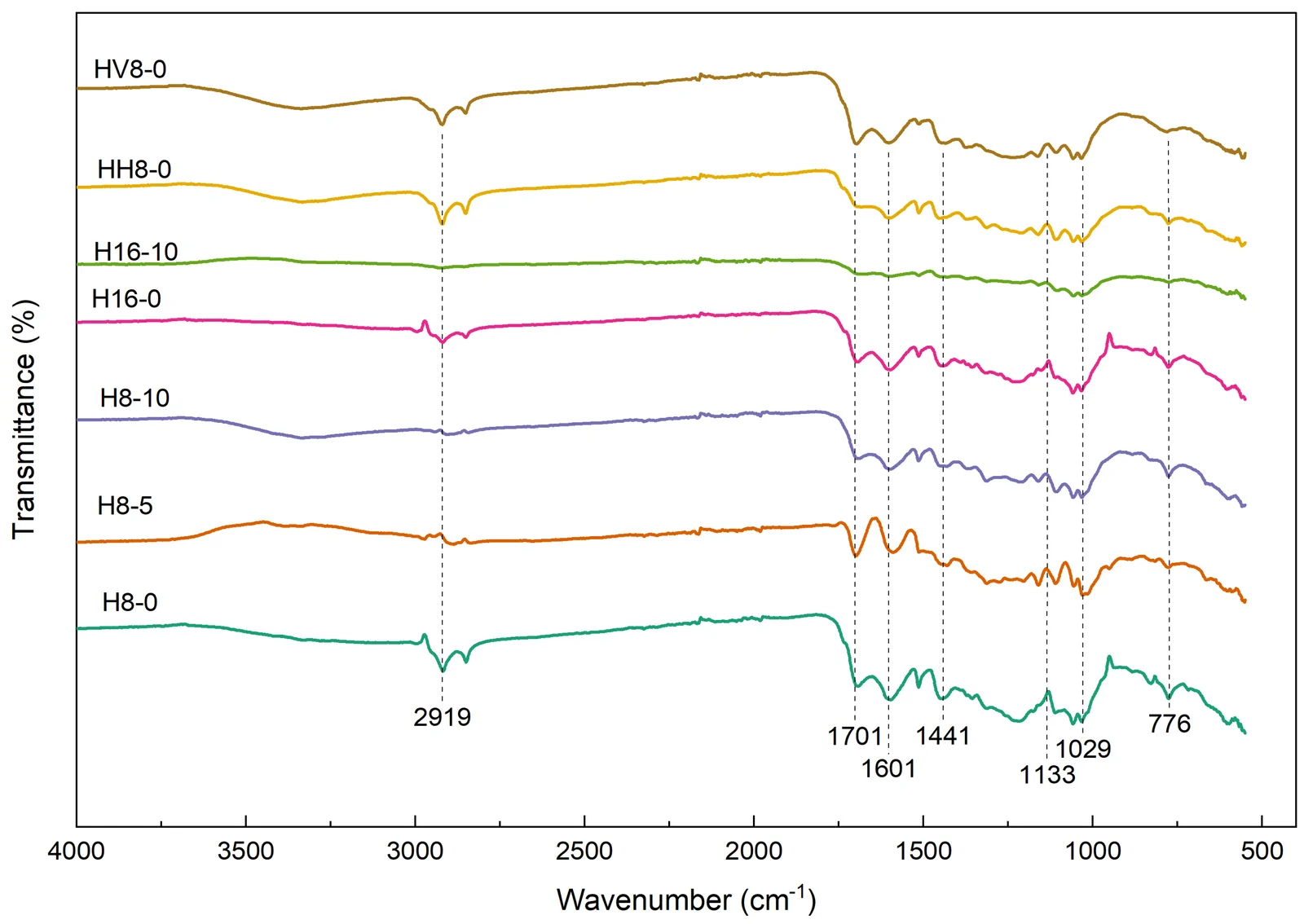
FTIR spectra of different hydrochars obtained under various conditions of the HTC process using agave leaves and tequila vinasse as residual biomass. H8-0 (hydrochar from leaves and vinasse with 8 h and 0% acid), H8-5 (hydrochar from leaves and vinasse with 8 h and 5% acid), H8-10 (hydrochar from leaves and vinasse with 8 h and 10% acid), H16-0 (hydrochar from leaves and vinasse with 16 h and 0% acid), H16-10 (hydrochar from leaves and vinasse with 16 h and 10% acid), HH8-0 (hydrochar from agave leaves and water with 8 h and 0% acid), and HV8-0 (hydrochar from vinasse with 8 h and 0% acid).

The FTIR analysis reveals the functional groups present in the different hydrochars (Figure 2). In the 3700–3000 cm^−1^ wavelength range, the hydroxyl group (-OH) is observed, except in the leaf hydrochars combined with 16 h vinasse at different catalyst concentrations. In the 3000–2900 cm^−1^ wavelength range, the aliphatic group (C–H) is present in all hydrochars. At 2360 cm^−1^, a signal from the C=C group is detected. While the characteristic band of carboxyl groups typically appears in the 1700–1730 cm^−1^ range, it shifts slightly to 1680–1710 cm^−1^ when conjugated with aromatic rings (Ref). A similar shift is observed in the spectra of the hydrochar samples reported in this study, owing to the presence of lignin, the dehydration of cellulose and hemicelluloses, and the subcritical water reaction conditions [31]. At 1690–1735 cm^−1^, the carboxyl C=O group is present associated with aromatic groups, although the signal in HH8-0 is very weak; at 1550–1650 cm^−1^ region, a band is observed that can be assigned to carboxyl (COOH) groups and aromatic C=C bonds, which arise from the dehydration and condensation reactions of carbohydrates (cellulose and hemicellulose) solubilized in the hydrothermal treatment [32]. At 1513 cm^−1^, the aromatic C=C group is present and found in all hydrochars except HV8. At 1460 cm^−1^, the methylene group (CH_2_) is not detected in H16-10. The ester group (C-O) is present in all hydrochars and is observed in the 1290–950 cm^−1^ wavelength range. Finally, the aromatic (C-H) group is present in all hydrochars at wavelengths of 860–724 cm^−1^.

These signals can be attributed to lignin-related bonds as well as to various vibrations in the cellulose and holocellulose structures. Therefore, if these signals weaken in some samples, it indicates that the structures are degrading, which is normal at temperatures above 200 °C [33,34].

**Figure 3:**
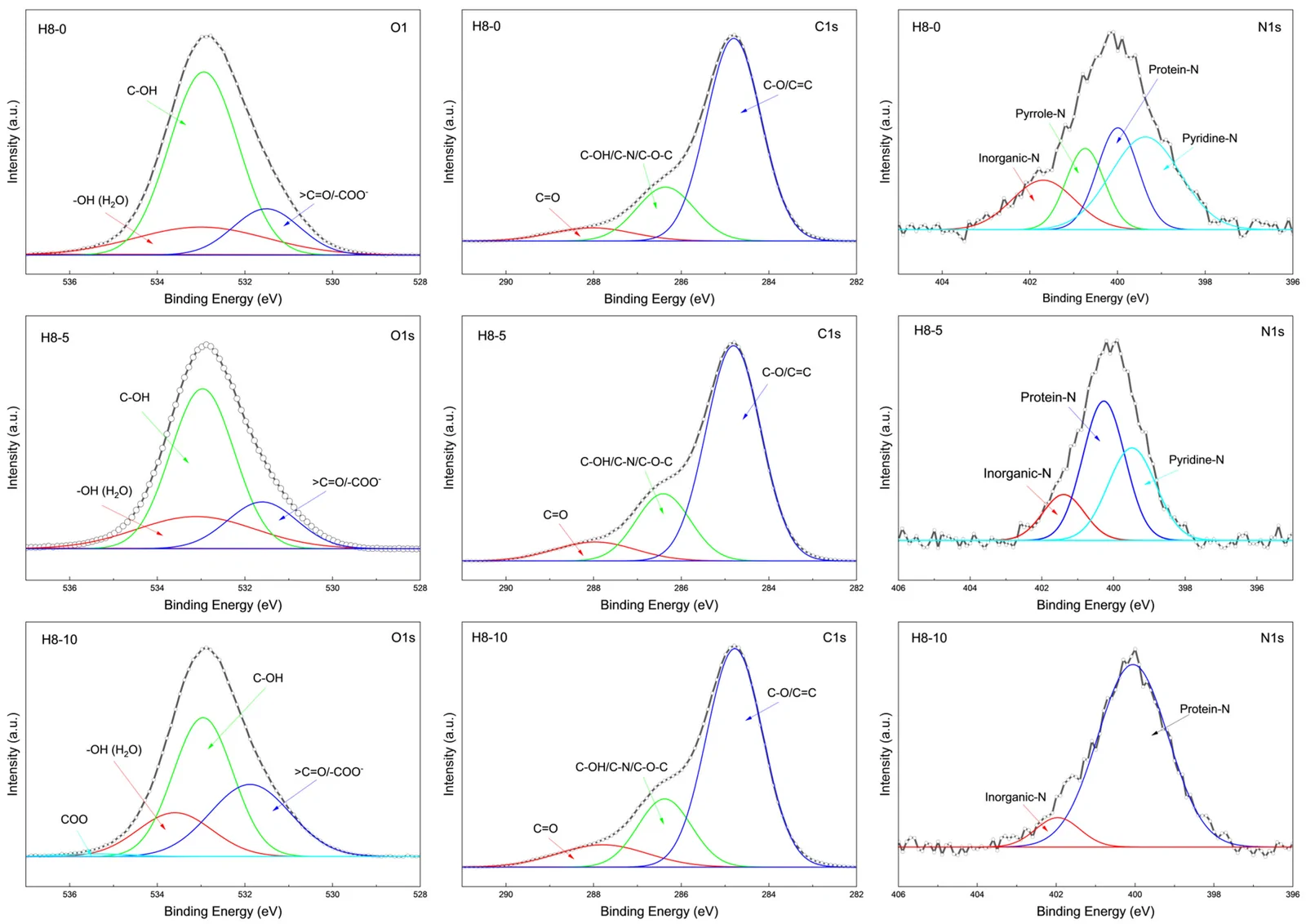
X-ray Photoelectron Spectroscopy (O1s, C1s, and N1s spectra) of different hydrochars obtained from the hydrothermal carbonization of agave leaves and tequila vinasse under various conditions. H8-0 (hydrochar from leaves and vinasse with 8 h and 0% acid), H8-5 (hydrochar from leaves and vinasse with 8 h and 5% acid), H8-10 (hydrochar from leaves and vinasse with 8 h and 10% acid).

**Figure 4:**
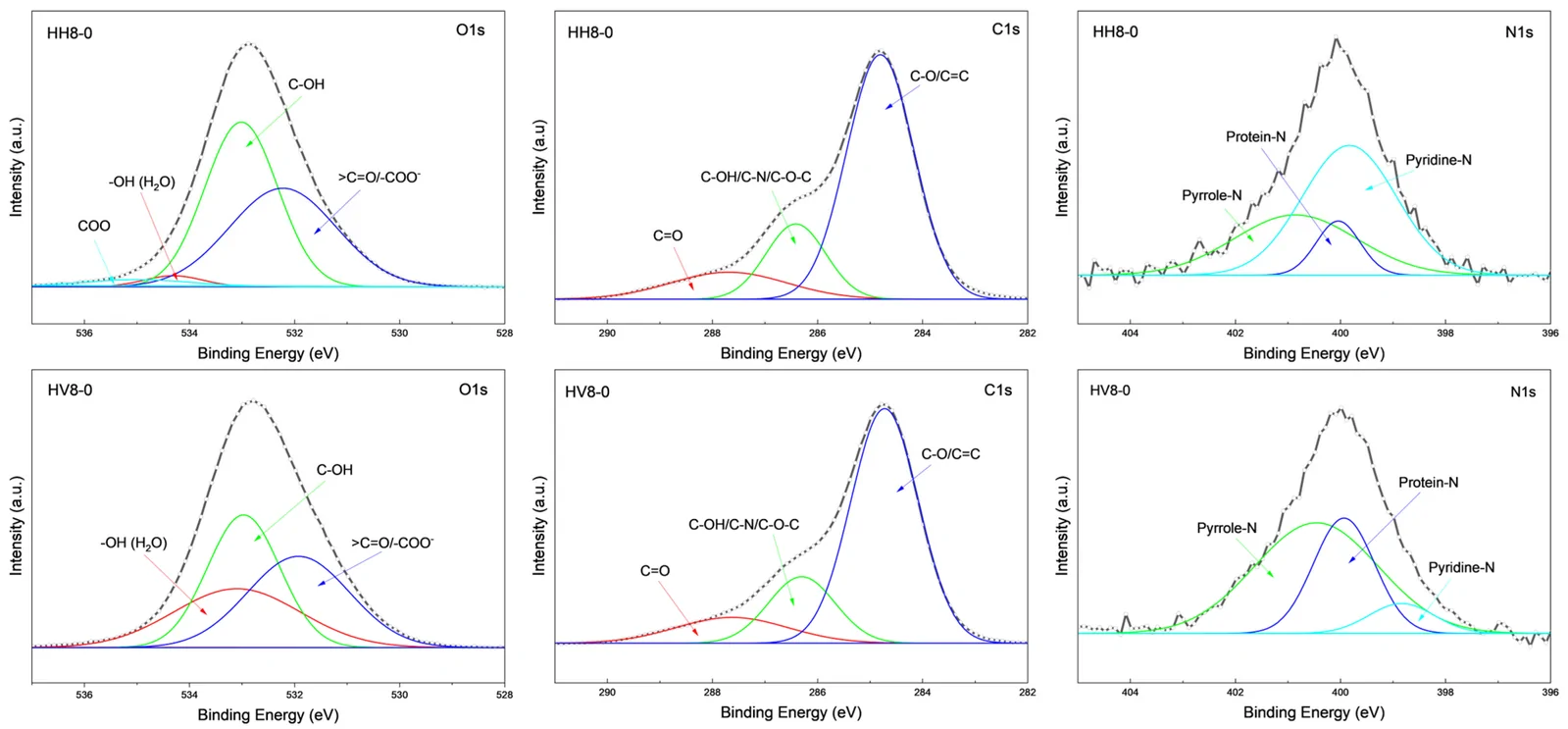
XPS deconvolution (O1s, C1s, and N1s spectra) of different hydrochars obtained under various conditions of the HTC process using agave leaf or tequila vinasse waste biomass. HH8-0 (hydrochar from agave leaves and water at 8 h and 0% acid) and HV8-0 (hydrochar from vinasse at 8 h and 0% acid).

The deconvolutions of the spectra were determined using the assignments and average atomic fractions (Figure 3 and Figure 4). For C1s, three curves were observed in all hydrochars, corresponding to peaks at 287.1 eV, 286.3 eV, and 284.7 eV, attributed to C=O, C-OH/C-N/C-O-C, and C-O/C=C, respectively. For the O1s spectrum, up to four curves were observed, with peaks at 534.6 eV (COO), 533.1 eV (-OH (H_2_O)), 532.51 eV (C-OH), and 531.3 eV (>C=O/-COO^−^). Finally, for the N1s spectrum, four main peaks were observed at 402 eV, 400.4 eV, 400 eV, and 398.8 eV, corresponding to inorganic nitrogen, pyrrolic nitrogen, protein nitrogen, and pyridine nitrogen, respectively [35,36,37].

According to the FTIR and XPS spectra, a higher concentration of carboxyl (-COOH) and hydroxyl (-OH) groups was observed at 8 h but decreased at 16 h. This trend is driven by continuous dehydration (H_2_O loss) and decarboxylation (CO_2_ release) under subcritical water conditions. Additionally, longer reaction times promote condensation and cross-linking reactions that consume these peripheral oxygenated groups, converting the aliphatic-rich intermediate into a more stable, polyaromatic hydrochar matrix with reduced O/C ratios. The depletion of -COOH and -OH groups with increasing residence time (8 h vs. 16 h) is attributed to progressive dehydration and decarboxylation reactions. Furthermore, extended reaction times favor secondary condensation and polymerization, consuming surface oxygenated groups to form a more aromatic and hydrophobic carbon structure [38].

The functional groups present on the surface of hydrochars may play an important role in their proposed use as soil conditioners. Carboxyl, hydroxyl, and carbonyl groups are responsible for changes in soil chemical activity, specifically in Cation Exchange Capacity (CEC). Carboxyl groups can generate negative charges that retain certain cations in the soil, even preventing them from leaching into the subsoil [39]. On the other hand, hydrogen bonding can be achieved thanks to the alcohol (C-OH) and ether (C-O-C) groups, improving the physical structure and its water-retention potential [40]. Likewise, the organic-mineral and aromatic nitrogen groups are key to meeting the energy demands of soil microorganisms, with rapid availability (inorganic and protein nitrogen) or to the stability and durability that hydrochar will have in the soil (pyrrole and pyridine nitrogen) and its contribution to long-term nitrogen storage [41].

**Figure 5:**
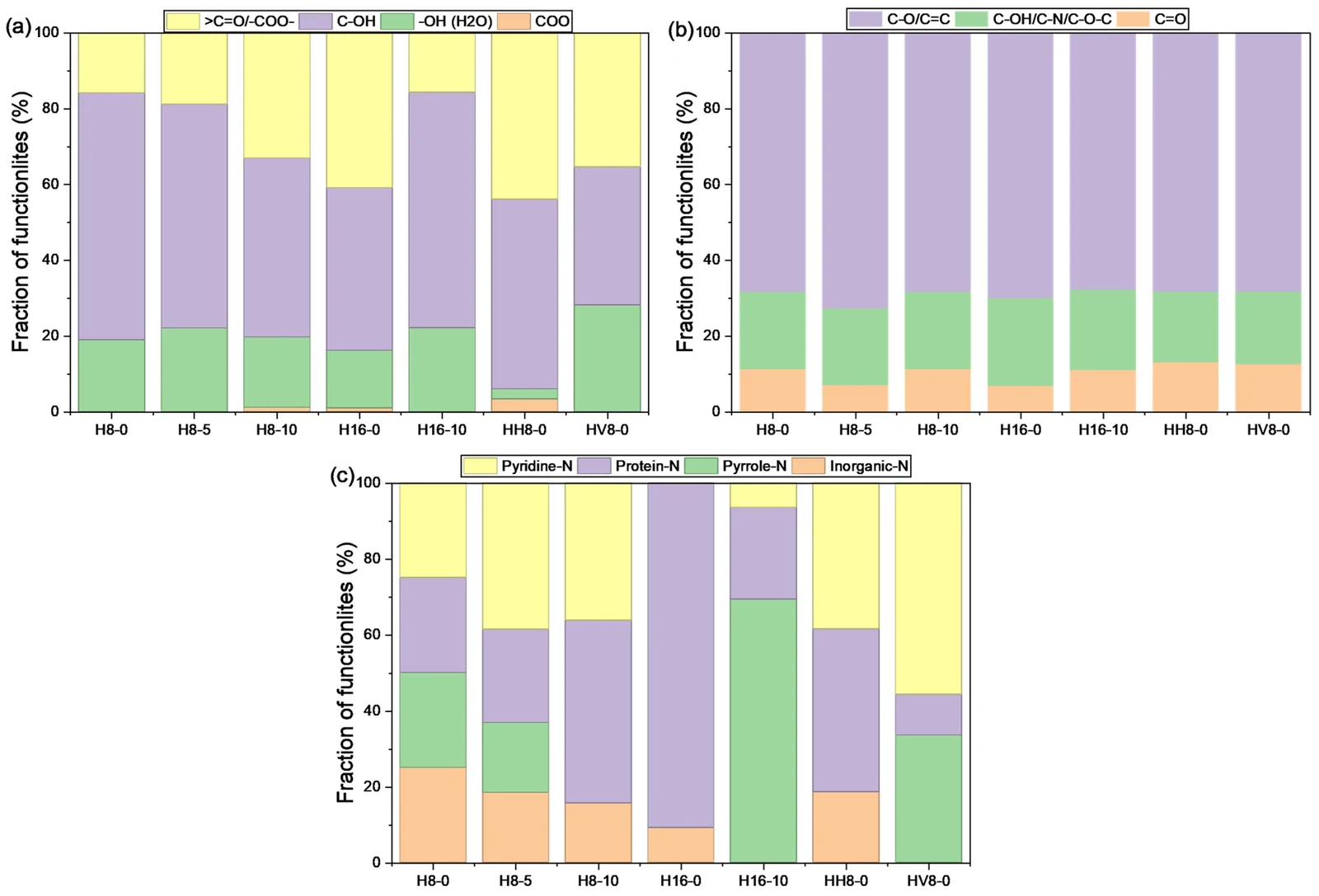
Relative functional content of O (**a**), C (**b**), and N (**c**) in different hydrochars obtained under various conditions of the HTC process using agave leaves or tequila vinasse as waste biomass. H8-0 (hydrochar from leaves and vinasse with 8 h and 0% acid), H8-5 (hydrochar from leaves and vinasse with 8 h and 5% acid), H8-10 (hydrochar from leaves and vinasse with 8 h and 10% acid), H16-0 (hydrochar from leaves and vinasse with 16 h and 0% acid), H16-10 (hydrochar from leaves and vinasse with 16 h and 10% acid), HH8-0 (hydrochar from agave leaves and water with 8 h and 0% acid), and HV8-0 (hydrochar from vinasse with 8 h and 0% acid).

Figure 5 shows changes in the content of the different hydrochar compounds. In some cases, the greatest variation was observed in the presence or absence of the functional groups COO, inorganic nitrogen, and pyrrole nitrogen. The alcohols decreased as the citric acid dose increased. In contrast, inorganic nitrogen is absent in H16-0 and in the hydrochars derived from raw materials HH8-0 and HV8-0. When applied to soil, the compounds described may change their structure and function through interactions with soil functional groups and microbial activity [42].

### 2.5. Microstructural Analysis of the Hydrochars

**Figure 6:**
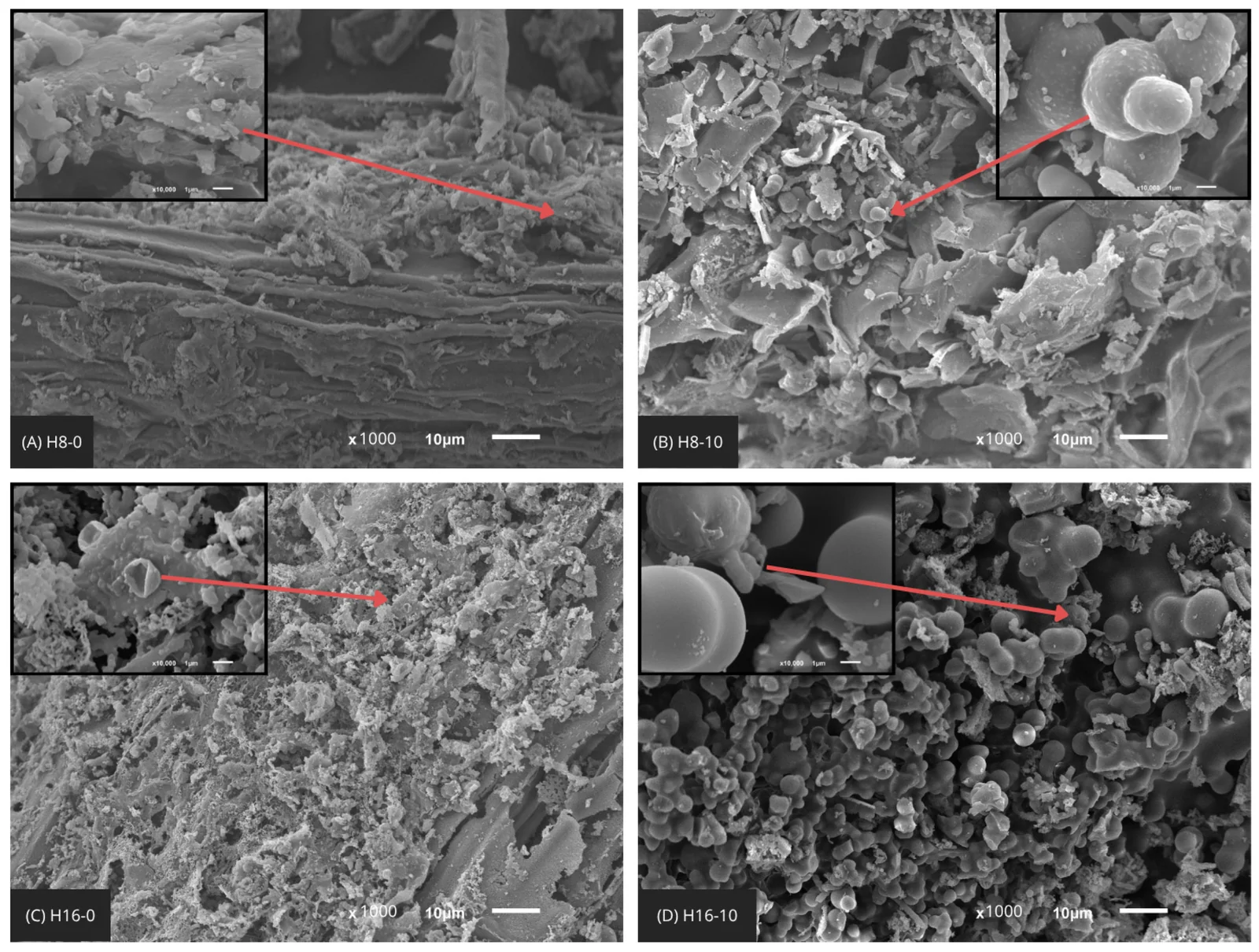
Micrographs of hydrochars obtained under different conditions of the HTC process of agave leaf and tequila vinasse waste biomass. H16-0 (hydrochar from leaves and vinasse with 16 h and 0% acid), H16-10 (hydrochar from leaves and vinasse with 16 h and 10% acid).

**Figure 7:**
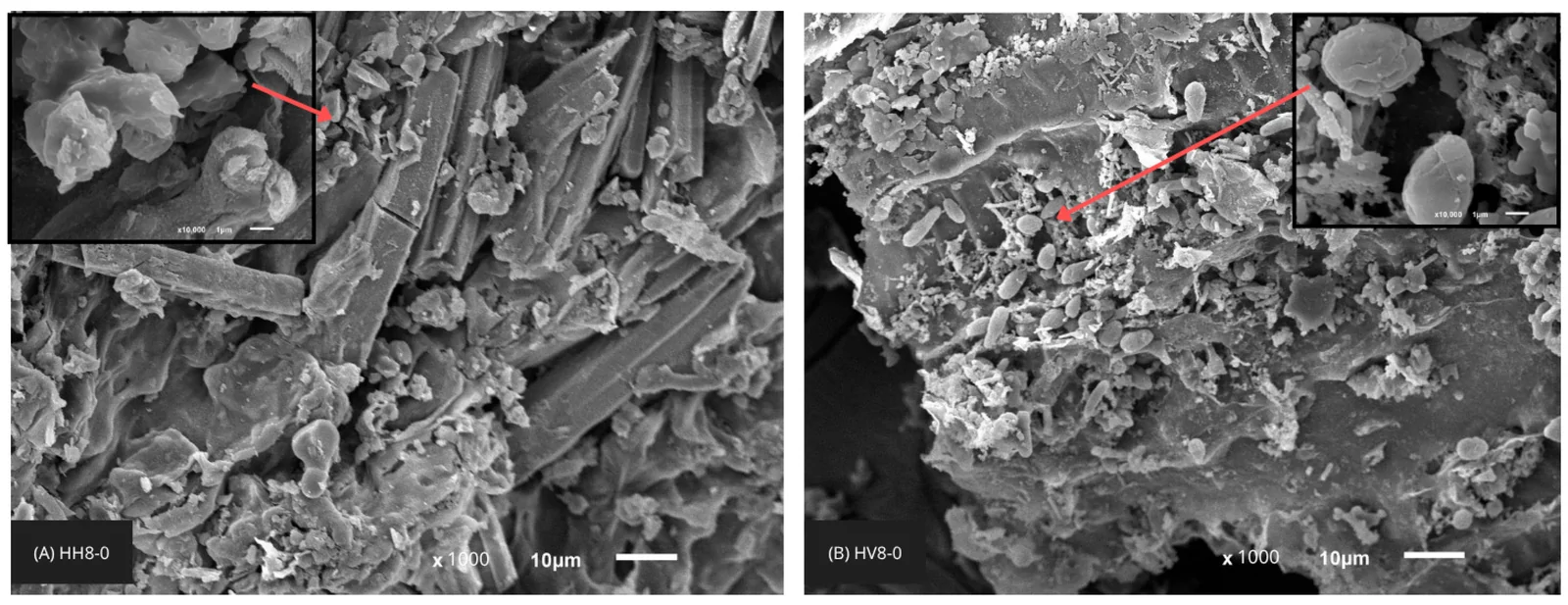
Micrographs of different hydrochars obtained under different conditions of the HTC process using agave leaf (**A**) and tequila vinasse (**B**) waste biomass. HH8 (Leaf Hydrochar) at 8 h. HV8 (Vinasse Hydrochar) at 8 h.

Figure 6 and Figure 7 show the microstructures of the hydrochars under different conditions in the HTC process. It can be observed that H8-0 (Figure 6A) has a corrugated laminar structure, which is due to the low degradation of lignin [43]. On the other hand, greater degradation is observed when 10% citric acid is used as a catalyst in the process (Figure 6B).

The hydrochars processed for 16 h (Figure 6C,D) exhibit a structure with more spherical formations resulting from the degradation of carbohydrates found primarily in the leaves (lignin, cellulose, and holocellulose); thus, it is inferred that longer reaction times will result in a more widespread distribution of these structures [44]. Furthermore, temperature influences the structure of the hydrochar; the shorter the retention time in the HTC process, the less likely this type of structure is to form, as can be seen in Figure 6A, since time controls the polymerization and hydrolysis processes and also influences the diameter of the spheres [45].

Finally, in Figure 7, the structural changes across the different initial biomasses can be observed. In the case of HH8-0, the structure is laminar and barely corrugated, although the formation of spheres begins to be observed. Compared to H8-0, the use of vinasse is confirmed to act as a catalyst, increasing the porosity of the hydrochar.

Due to its porous structure and oxygen-containing functional groups, hydrochar from agave leaves and vinasse can improve soil quality through adsorption and other effects during application [46]; furthermore, due to its nutrient content, it can serve as a suitable habitat that promotes the growth and survival of soil microorganisms [47].

### 2.6. Characterization of the Leachate

Table 2 shows the characteristics of the leachates obtained from the HTC process. It can be observed that combining leaves with vinasse in the HTC process improved the pH of the original vinasse, increasing the initial pH from 3.8 to 4.4. The highest pH values were obtained in hydrochars HH8 and HH16, made from leaves alone and without a catalyst (4.8, 4.7), followed by hydrochars made from a combination of leaves and vinasse at 12 h, 8 h, and 16 h. The lowest pH values were observed in vinasse hydrochars, with a pH of 3.8, indicating that the pH of the raw material is maintained; therefore, adding agave leaves during the hydrothermal carbonization process improves this characteristic. Li et al. [48] observed that, in a manure hydrochar, the pH increases as the temperature rises.

It was observed in the vinasse hydrochar without a catalyst (HV8) that the HTC process reduces the initial Total Solids (TS) content of the vinasse (32.6 g/L) to 15–10 g/L, which equates to a reduction in TS of up to 70%. Some alternatives, such as the application of a cationic polyacrylamide flocculant to various vinasses with TS contents ranging from 24 to 40 g/L, reduce this content by up to 30% [49]. On the other hand, when using vinasses with a TS content of 29.9 g/L as a substrate in an anaerobic system (UASB) connected in series, TS levels can be reduced by up to 50% in stage V of said process [50]. In the leachate resulting from the HTC process using leaves and vinasse, the contaminant parameters of the raw vinasse change: the pH increases and the total solids decrease. It is also observed that the resulting pH is similar regardless of whether acid or vinasse is used; this is primarily because tequila vinasse provides sufficient natural acidity to catalyze the reaction. Therefore, the addition of citric acid serves mainly to improve kinetics and yield without drastically lowering the final pH any further [51].

These changes would represent a lower environmental impact for final disposal.

H8-0 (hydrochar from leaves and vinasse with 8 h and 0% acid), H8-5 (hydrochar from leaves and vinasse with 8 h and 5% acid), H8-10 (hydrochar from leaves and vinasse with 8 h and 10% acid), H16-0 (hydrochar from leaves and vinasse with 16 h and 0% acid), H16-10 (hydrochar from leaves and vinasse with 16 h and 10% acid), HH8-0 (hydrochar from agave leaves and water with 8 h and 0% acid), and HV8-0 (hydrochar from vinasse with 8 h and 0% acid).

### 2.7. Cost–Benefit Analysis of a Hydrochar Production Plant

**Figure 8:**
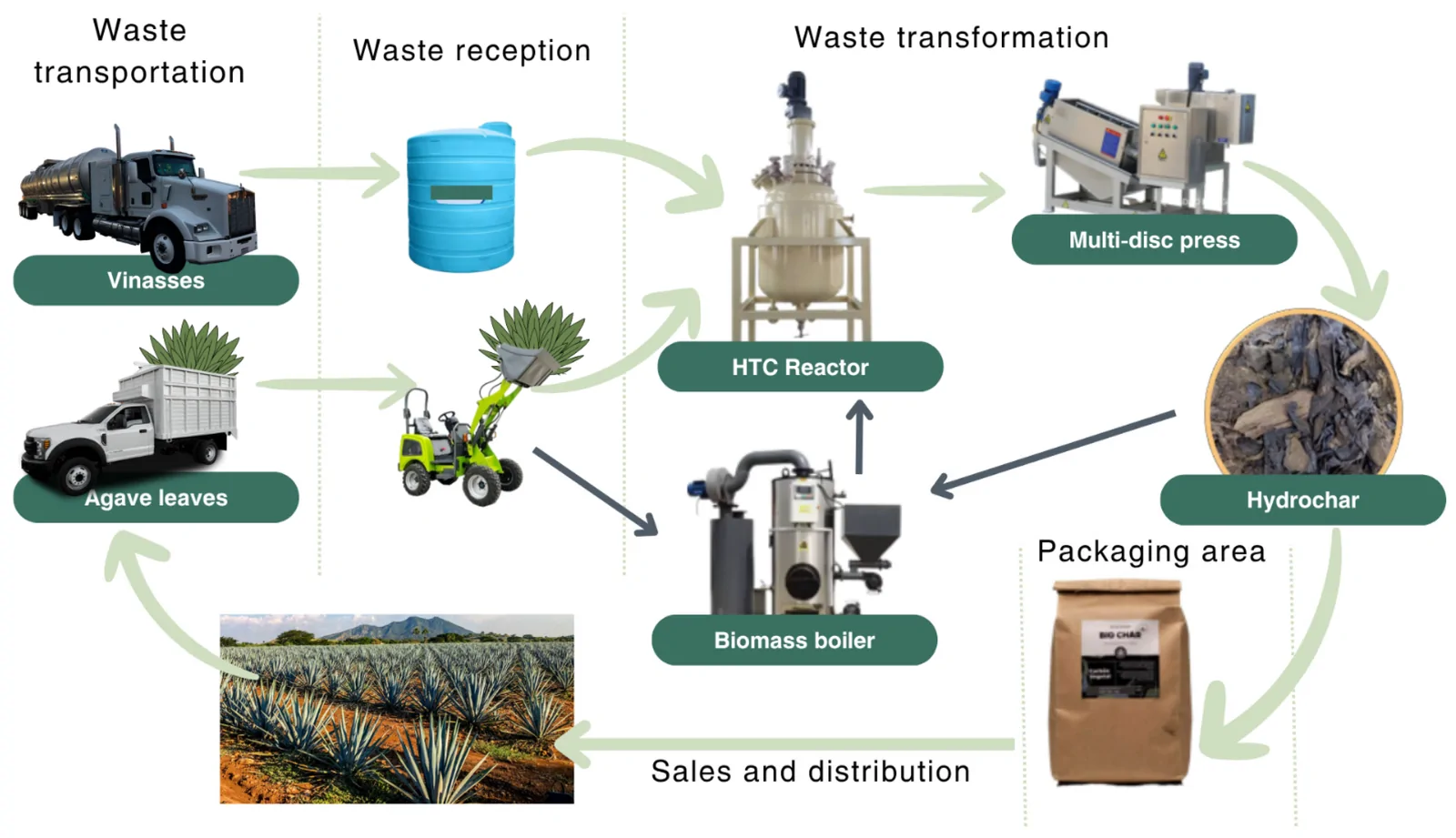
Proposed biorefinery scheme for tequila vinasse and agave leaves for the tequila industry in Mexico.

The following is a cost–benefit analysis of a hydrochar production plant hypothetically located in Jalisco, Mexico. Figure 8 illustrates the processing stages for these waste streams.

A hydrothermal carbonization (HTC) biorefinery is proposed, hypothetically located in Jalisco, Mexico, to take advantage of the high availability of raw materials and thereby reduce transportation costs. The facility will feature a 4000 L reactor capable of processing 6400 L of vinasse and 3.5 tonnes of agave leaves daily to produce 300 kg of hydrochar. A multi-disk press is proposed for solid–liquid separation, eliminating the need for dryers, alongside a biomass boiler to reduce energy costs. The solid hydrochar product will be sent to the packaging area for sale and distribution. The intention is that the gas produced in the HTC process will be used as an energy source and that the liquid fraction will be used as an agricultural input, although a complete characterization of this fraction is still needed. Table 3 presents a breakdown of the total costs (in USD) required as the initial investment to establish the production plant, the initial investment costs are detailed in Table S2 of the Supplementary Material. These costs are based on 2026 prices.

### 2.8. Cost–Benefit Ratio (C/B)

Based on the estimated data in Table 4, the monthly operating costs required to calculate the C/B ratio are shown.

C/B Ratio = 24,750.00 / 18,165.00≈1.36

This means that for every $1 MXN spent on operating the HTC plant, $1.36 in gross revenue is generated. The value of this ratio must be >1, so in conclusion, this figure is excellent.

### 2.9. Return on Investment (ROI)

The data required to calculate the *ROI* are as follows:

Total investment: $44,741.00

Monthly net profit: $18,165.00

$$ROI\;\left(months\right)=44,741.00/18,165.00\approx 2.46\;months$$

This means that, in an ideal scenario with 100% sales, the time it would take for the project to recover the total investment (total costs plus initial expenses) would be 2.5 months, indicating financial viability and low investment risk.

### 2.10. Net Present Value (NPV)

Projection assumptions for the calculations:

Project horizon (*n*): 5 years

Annual Net Cash Flows (FC): $217,684.80

Initial Investment (I_0_): $44,741.00

Discount Rate (*r*): 15%

NPV=−44,741.00 + 729,713.21 ≈ $768,872.50 USD

A positive and high Net Present Value (*NPV*) indicates that the project recovers the investment and covers the opportunity cost of capital (15%). Furthermore, it generates significant additional profit on a present-value basis.

### 2.11. Internal Rate of Return (IRR)


IRR≈480

Given that *NPV* = 0, the rapid recovery of the investment, and the annual cash flow, the IRR is extremely high. Therefore, the project has a very good internal rate of return and may be financially superior to alternative investments.

## 3. Materials and Methods

### 3.1. Obtaining Materials for Hydrochar Production

Two types of agro-industrial waste were used in hydrochar production: agave leaves and tequila vinasse.

Leaves were collected from a field of 5-year-old *Agave tequilana* Weber var. azul that had recently been harvested in the municipality of El Arenal, Jalisco (20°45′26.9532″ N, 103°40′7.1418″ W), within the Denomination of Origin (DOT) area for tequila. After collection, the leaves were cut into 10–15 cm pieces and frozen for subsequent characterization and processing. In addition, raw vinasse (without any prior treatment) from a diffuser (a process for extracting sugars by diffusion) was used; this was donated by a tequila company located in Guadalajara, Jalisco, and was stored in hermetically sealed containers at room temperature until use.

### 3.2. Chemical Characterization of Agave Leaves and Tequila Vinasse

The moisture content of the leaves was determined by the difference in wet and dry weights, with the samples subjected to 105 °C for 12 h in an oven; subsequently, the moisture content was calculated as

(PH−PS)/PH∗100,

where

*PH*: wet weight

*PS*: dry weight

The determination of total extracts from the leaves was performed according to the TAPPI T264 om-12 [52] method. Briefly, 10 g of leaves were extracted with an ethanol/toluene mixture (1:2) for 24 h, then washed with ethanol under vacuum filtration for 8 h, and finally with distilled water for 1 h. The residues were then washed, oven-dried at 105 °C, and the dry residue was weighed. The percentage of each fraction was calculated relative to the original dry pulp mass; the sum of all these fractions corresponds to the total extract content.

The holocellulose content in the leaves was determined according to TAPPI method T-249 sm [52] by treating the extract-free biomass with chlorine dioxide generated in situ by adding sodium chlorite to an acetic acid medium. This treatment solubilizes lignin, leaving a solid containing all the present polysaccharides.

Klason lignin in the leaves was quantified using the TAPPI 222 om-11 [52] method; a two-step quantitative acid hydrolysis was performed. In the first stage, 72% sulfuric acid was used to hydrolyze the polysaccharides into oligosaccharides; the oligosaccharides then precipitated as an insoluble fraction in the acidic medium. In the second stage, 4% sulfuric acid was used to break down the oligomers into monosaccharides, leaving a solid fraction consisting of lignin.

To characterize the vinasses, total solids (TS) were determined by measuring 10 mL of sample and heating it to 105 °C until a constant weight was achieved.

The Horiba Scientific^®^ F-71 pH meter was used to determine the pH of the vinasses in accordance with NMX-AA-008-SCFI-2016 [53] and the electrical conductivity in accordance with NMX-AA-093-SCFI-2000 [54]. The electrode was inserted directly into the sample prepared at a 2:1 vinasse-to-distilled-water ratio, and the measurement was performed in triplicate.

### 3.3. Standardization of Hydrochar Production and Yield

To evaluate the HTC process conditions, a 5 L capacity JAYME rotary reactor (Deutsch & Neumann GmbH, Hennigsdorf, Germany) was used. To avoid biomass variation between treatments, the agave leaves were weighed, and the volume of tequila vinasse was measured to fill 80% of the reactor’s capacity (2200 g of leaves and 4 L of vinasse).

To establish the treatments, the concentration of citric acid (0%, 5%, and 10%) as a catalyst and reaction times (8 h, 12 h, and 16 h) were varied. Two additional treatments were added by varying the initial biomass, as described below: Agave leaves, water, no catalyst, 8 h (HH8) and tequila vinasse, no water, no catalyst, 8 h (HV8).

The temperature was maintained at a constant 200 °C under self-generated pressure. Once the HTC process was complete, phase separation was achieved by vacuum filtration, and the hydrochar was then dried in an oven for 24 h at 105 °C [55].

The yield was calculated using the following formula:

$$R=\left(Bi-Hch\right)\cdot 100$$

where

*R* = yield, Bi = dry weight of the initial biomass, and Hch = final dry weight of the hydrochar. The initial and final biomass of the HTC process is indicated in Table S1 of the Supplementary Material.

Of the eleven hydrochars obtained, only those that exhibited the highest yield or were produced under contrasting HTC conditions were selected for subsequent analysis and characterization, resulting in the following seven: H8-0 (hydrochar from leaves and vinasse with 8 h and 0% acid), H8-5 (hydrochar from leaves and vinasse with 8 h and 5% acid), H8-10 (hydrochar from leaves and vinasse with 8 h and 10% acid), H16-0 (hydrochar from leaves and vinasse with 16 h and 0% acid), H16-10 (hydrochar from leaves and vinasse with 16 h and 10% acid), HH8-0 (hydrochar from agave leaves and water with 8 h and 0% acid), and HV8-0 (hydrochar from vinasse with 8 h and 0% acid).

To characterize the seven selected hydrochars, X-ray fluorescence (XRF) analysis was performed using a Genius 5000 XRF^®^ portable analyzer (Jiangsu Skyray Instrument Co., Ltd., Kunshan, China), a non-destructive method for determining elemental concentrations. The following elements were measured in mg/kg: K, Cu, Fe, Mo, Mn, Zn, Ni, Sn, Ga, Rb, Sr, Y, Zr, Nb, Ti, V, Al, Cr, as well as elements not detected in the samples: Co, As, Ag, Sb, Hg, Pb, Th, U.

In addition, to identify the functional groups present in the sample, Fourier transform infrared spectroscopy (FTIR) was used; the samples were analyzed in the range from 4000 cm^−1^ to 500 cm^−1^ with a resolution of 4 cm^−1^; the equipment used was an Alpha-T FTIR-ATR from Bruker Optics^®^ (Bruker Optics GmbH & Co. KG, Ettlingen, Germany), performing 23 sweeps per sample.

Characterization was also performed using a K-Alpha spectrometer (Thermo Scientific, East Grinstead, UK). The chamber pressure during the analysis was 2.2 × 10^−7^ mbar. A monochromatic Al Kα (1486.68 eV) X-ray source was used with an analysis radius of 400 μm. The survey analysis was performed with a step width of 150 eV and 1 eV steps, while the high-resolution spectra were obtained with a step width of 50 eV and 0.1 eV steps. Elements were automatically identified and quantified in the survey using Thermo Advantage software (v6.9, Thermo Fisher Scientific). The deconvolution of the spectra was performed in the software Origin Pro 2025b (R), making a baseline adjustment and using the following parameters on the Binding energy scale: O1 (537-528), C1 (291-282) and N1 (405-396).

The pH and electrical conductivity of the hydrochars in a 2:1 suspension (distilled water: hydrochar) were determined in triplicate using a LAQUA F-71 benchtop pH meter (Horiba, Ltd., Kyoto, Japan) (NMX-AA-008-SCFI-2016) and (NMX-AA-093-SCFI-2000). In addition, the total carbon and nitrogen content of the samples was determined by flash combustion (Modified Dumas Method) with thermal conductivity detection using a Thermo Scientific FlashSmart Elemental Analyzer.

To examine the morphology of the different coals obtained, scanning electron microscopy (SEM) was performed on a high-resolution TESCAN MIRA 3LMU microscope. Sample preparation involved immobilizing the charcoal on copper tape and coating it with gold for 20 s. Additionally, energy-dispersive X-ray spectroscopy (EDS) was performed to confirm the elements present in the charcoal.

### 3.4. Financial Analysis of Hydrochar Production with Agricultural Potential

An economic cost–benefit analysis was conducted based on the installation of a biorefinery for the production of hydrochar with potential for agricultural use. The following equations were used [56]:

Benefit−Cost Ratio=Gross income/Monthly net profit

Return on Investment= Total investment/Monthly net profit

For the analysis of Net Present Value (*NPV*) and Internal Rate of Return (*IRR*), a 5-year projection (*n*) was made, and a discount rate (*r*) of 15% was used, as this is a conservative rate for the agro-industrial sector.

$$NPV=-I_{0}+\sum_{t=t}^{n}\frac{FCt}{\left(1+r\right)t}$$

$$IRR:0=-I_{0}+\sum_{t=t}^{n}\frac{FCt}{\left(1+TIR\right)t}$$

where *I*_0_ is the initial investment; *n* corresponds to the project horizon; *FC* is the Annual Net Cash Flow; and *r* is the Discount Rate.

## 4. Conclusions

The conversion of tequila vinasse and agave leaves via hydrothermal carbonization yielded a value-added hydrochar with potential application as a soil amendment; therefore, soil and plant tests are suggested to evaluate its effects.

On the other hand, the 8 h hydrothermal carbonization (HTC) process, compared with longer durations (12 and 16 h), produced hydrochar with the yield and characteristics necessary to obtain a product with structural and chemical properties potentially suitable for soil application. The acidic nature of the vinasse positively influences the HTC process, primarily increasing the process yield. The addition of vinasse reduces the need to add acidic agents and water to the process, while enabling the conversion of another highly polluting waste product from the tequila industry. The cost–benefit analysis of the process indicates that incorporating HTC represents a viable alternative for the recovery of tequila waste under a circular economy approach, with the potential to generate a soil conditioner and contribute to climate change mitigation.

## Figures and Tables

**Table 1:** Chemical characterization of the raw materials and hydrochars selected based on yield and contrasting conditions of the HTC process.

Sample	pH	EC (dS/m^−1^)	C	N	C/N	K	Ca	F	Mn	Ni	Zn	Mo
			%			ppm						
Agave leaves	5.3	x	x	1.17	x	814.6	575.38	358.26	53.22	-	-	6.44
Tequila vinasse	3.8	1.6	-	0.25	x	183.95	463.66	972.12	48.07	-	-	5.09
H8-0	5.3	0.8	59.81	1.825	33	805.9	418.97	83.85	81.22	16.44	32.37	2.34
H8-5	5.4	1.0	60.87	1.903	32	-	563.01	621.20	66.46	-	26.65	6.0
H8-10	5.5	0.7	61.48	1.978	31	385.33	103.58	747.60	57.33	-	2.88	6.48
H16-0	5.0	0.7	60.84	1.634	37	977.28	158.59	963.99	59.07	-	-	5.46
H16-10	5.3	0.7	59.87	2.104	28	-	685.90	904.74	68.59	28.20	2.32	0.39
HH8-0	5.5	0.7	58.97	1.670	35	85.67	334.63	891.25	53.30	94.33	94.33	5.46
HV8-0	5.0	1.0	62.24	2.263	28	16.78	845.59	729.33	56.24	41.09	20.32	4.09

(-) Not detected, (x) Not determined. H8-0 (hydrochar from leaves and vinasse at 8 h and 0% acid), H8-5 (hydrochar from leaves and vinasse at 8 h and 5% acid), H8-10 (hydrochar from leaves and vinasse at 8 h and 10% acid), H16-0 (hydrochar from leaves and vinasse at 16 h and 0% acid), H16-10 (hydrochar from leaves and vinasse at 16 h and 10% acid), HH8-0 (hydrochar from agave leaves and water at 8 h and 0% acid), and HV8-0 (hydrochar from vinasse at 8 h and 0% acid).

**Table 2:** Chemical characterization of the leachates obtained under different conditions of the HTC process of agave leaf and tequila vinasse waste biomass.

Treatment	pH	EC (dS/m^−1^)	TS (g/L)
H8-0	4.4	1.3	25.0
H8-5	4.4	1.3	27.5
H8-10	4.4	1.3	20.0
H12-0	4.5	1.3	25.0
H12-5	4.5	1.2	25.0
H12-10	4.4	1.3	30.0
H16-0	4.3	1.4	25.0
H16-5	4.3	1.4	30.0
H16-10	4.4	1.3	25.0
HH8-0	4.6	1.2	15.0
HH12-0	4.7	1.2	15.0
HH16-0	4.8	1.0	10.0
HV8-0	3.8	1.7	10.0
HV16-0	3.5	1.7	15.0

EC: Electrical Conductivity, TS: Total Solids.

**Table 3:** Total costs for the initial investment of an agave leaf and tequila vinasse HTC plant with a production capacity of 9 t per month of hydrochar.

Concept	Cost (USD)
Infrastructure	$1375
Waste transportation	$300
Waste reception	$7595
HTC production	$14,146
Packaging and storage	$402
Sales and distribution	$14,700
Office	$990
Staff	$3900
Other inputs	$560
Stationery	$773
Total	$44,741

**Table 4:** Monthly operating costs of an agave leaf and tequila vinasse HTC plant.

Concept	Value (USD)
Gross Monthly Income	$24,750
Monthly Operating Costs	$6585
Monthly Net Profit	$18,165

## Data Availability

The original contributions presented in this study are included in the article/Supplementary Material. Further inquiries can be directed to the corresponding author.

## Supplementary Materials

The following supporting information can be downloaded at: https://www.mdpi.com/article/10.3390/molecules31183173/s1, Table S1: Inicial and final biomass of the HTC process, Table S2: Breakdown of initial investment costs for establishing a biorefinery.
