# Supplementary material for: Production and Physicochemical and Structural Characterization of a Hydrochar Derived from Agave Leaves and Tequila Vinasse with Potential Agricultural Applications
Source: Molecules. 2026 Sep 10;31(18):3173. doi: 10.3390/molecules31183173 (PMC13609801; doi:10.3390/molecules31183173)
Supplement: Supplementary file 1 [file molecules-31-03173-s001.zip › molecules-4445644-supplementary.pdf]

Supplementary material

Table S1. Inicial and final biomass of the HTC process.

Treatment	Reactor capacity	Inicial biomass (g)	Final biomass hydrochar (g)	Yield % Hydrochar	Total Solids (TS) lecheate (g/L)	Biomass balance (g)
H8-0	1 L	111.57	49.7	**44.55**	**25.0**	**36.87**
H12-0	5 L	557.86	216.3	**38.77**	**25**	**241.56**
H16-0	5 L	557.86	227.02	**40.69**	**30**	**210.84**
H8-5	1 L	111.57	43.62	**39.10**	**27.5**	**40.45**
H12-5	1 L	111.57	43.27	**38.78**	**25**	**43.3**
H16-5	1 L	111.57	44.29	**39.70**	**30**	**37.28**
H8-10	1 L	111.57	46.47	**41.65**	**20**	**45.1**
H12-10	1 L	111.57	41.42	**37.12**	**30**	**40.15**
H16-10	1 L	111.57	38.33	**34.35**	**25**	**48.24**
HH8-0	1 L	85.49	41.1	**48.07**	**15**	**29.39**
HV8-0	1 L	26.08	9.03	**34.64**	**10**	**7.05**

Biomass calculations were performed on dry basis

Biomass balance was calculated as: $Inicial\ biomass - (Final\ biomass\ hydrochar + TS\ lecheate)$. The rest is attributed to gases.

Table S2. Breakdown of initial investment costs for establishing a biorefinery.

Material	Description	Amount	Payment periodicity	Cost (MXN)	Supplier
Infrastructure					
Warehouse for rent	15x12 m warehouse in la Venta del Astillero, Jal		Monthly	$25.000,00	
Electricity service			Monthly	$1.500,00	
Water service			Monthly	$1.000,00	
**TOTAL**				**$27.500,00**	
Waste transportation					
Pick up	Gasoline		Monthly	$6.000,00	
**TOTAL**				**$6.000,00**	
Waste reception					
Cistern 10,000 L	Cistern Aquaplas 10,000 L Tricapa.	1 pza	Only payment	$19.900,00	Aquaplas
Transport pipes	1.5 inch suction hose	20 mt	Only payment	$2.000,00	Mercado libre

Forklift	Gasoline skid, steed loader FLAND 2024. 1m shovel 200 kg load capacity	1 pza	Only payment	$130.000,00	Mercado libre
**TOTAL**				**$151.900,00**	
HTC Process					
Reactor with rotator 1,000 L	Stainless Steel reactor 5 KL, Hidrothermal carbonization 4000 L	1	Only payment	$230.000,00	Laizhou Yisu Machinery Co., Ltd.
Boioler	Biomass boiler 200 Kg	1	Only payment	$24.300,00	Xinxiang Xinda Boiler Co.,ltd.
Filtres	Multidisc filter press, for manure sludge dewatering	1	Only payment	$28.188,00	Qingdao Dongmao Environmental Protection Equipment Co., Ltd.
Pipes	PVC 4" 6m	2	Only payment	$439,00	Grupo ferretero el campesino
**TOTAL**				**$282.927,00**	
Packaging and storage					
Packing tables	Stainless Steel table 110*60*90 cm	2	Only payment	$5.974,00	MINDEJAL
40 Kg Sacales	Digital scale 40kg. Gutstark	2	Only payment	$938,00	Mercado libre
Pellets	Wooden pallets EuroPalet. 1.20m x 80cm x 13.5cm	5	Only payment	$1.125,00	Mercado libre
**TOTAL**				**$8.037,00**	
Sales and Distribution					
Pick-up truck	JAC Frison 2021 4p Frison T6 L4/2.0/T Man.	1	Only payment	$289.000,00	Seminuevos Plasencia
Gasoline			Monthly	$5.000,00	
**TOTAL**				**$294.000,00**	
Office					
Computer	HP All-in-One 22-dg0005la, Desktop	2	Only payment	$11.000,00	Amazon
Desk	Desk L type	2	Only payment	$5.400,00	OfficeDepot
Office chair	Office chair Coscorp Lancaster	2	Only payment	$3.400,00	OfficeDepot
**TOTAL**				**$19.800,00**	
Operators					
Manager	1 person	1	Monthly	$16.000,00	

HTC process operators	3 people	3	Monthly	$30.000,00	
Packaging and storage	2 people	2	Monthly	$20.000,00	
Sales and distribution	1 person	1	Monthly	$12.000,00	
**TOTAL**				**$78.000,00**	
Other supplies					
5 Kg bags	Kraft paper P/5 Kg	200	Monthly	$3.600,00	Mercado libre
10 Kg bags	Kraft paper P/10 Kg	100	Monthly	$5.048,00	Mercado libre
25 Kg bags	Kraft paper P/25 Kg	50	Monthly	$2.544,00	Mercado libre
**TOTAL**				**$11.192,00**	
Starionery					
Trademark registration	For the review of a national trademark registration	1	Only payment	$2.458,00	IMPI
Trademark desing	Logo and website desing		Only payment	$9.000,00	
Marketing	Social media		Monthly	$4.000,00	meta
**TOTAL**				**$15.458,00**	
